# A novel autoantibody targeting calreticulin is associated with cancer in patients with idiopathic inflammatory myopathies

**DOI:** 10.1002/cti2.1195

**Published:** 2020-10-15

**Authors:** He Chen, Heng Yang, Qiu‐Xiang Cheng, Yong‐Peng Ge, Qing‐Lin Peng, Ya‐Mei Zhang, Gen‐Hong Cheng, Guo‐Chun Wang, Xin Lu

**Affiliations:** ^1^ Department of Rheumatology China‐Japan Friendship Hospital Beijing 100029 China; ^2^ Center for Systems Medicine Institute of Basic Medical Sciences Chinese Academy of Medical Sciences & Peking Union Medical College Beijing 100005 China; ^3^ Suzhou Institute of Systems Medicine Suzhou Jiangsu 215123 China; ^4^ Department of Microbiology, Immunology and Molecular Genetics University of California Los Angeles CA 90095 USA

**Keywords:** anti‐calreticulin autoantibodies, biomarkers, idiopathic inflammatory myopathy, malignancy

## Abstract

**Objectives:**

To investigate the prevalence and clinical significance of anti‐calreticulin autoantibodies (anti‐CRT Ab) in a large cohort of idiopathic inflammatory myopathy (IIM) patients.

**Methods:**

Sera from 469 patients with IIM, 196 patients with other connective tissue diseases, 28 patients with solid tumors and 81 healthy controls were screened for anti‐CRT Ab by enzyme‐linked immunosorbent assay using human recombinant CRT protein. Sera from 35 IIM patients were tested using an immunoprecipitation assay to confirm the presence of anti‐CRT Ab. Subsequently, IIM–cancer patients were identified and divided into new‐onset, remission and recurrent groups based on their cancer status. The relationships between anti‐CRT Ab levels and IIM disease activity were also investigated.

**Results:**

Serum anti‐CRT Ab was detected positive in 81 of the 469 (17.3%) IIM patients. Immunoprecipitated bands were observed at a molecular weight of 60 kDa corresponding to the CRT protein. The IIM patients with anti‐CRT Ab more frequently had cancers compared to the patients without anti‐CRT Ab. Moreover, the prevalence of anti‐CRT Ab differed according to the cancer status. The IIM patients with recurrent cancers had a much higher prevalence of anti‐CRT Ab than those with cancers in remission. Also, serum anti‐CRT Ab levels positively correlated with disease activity at baseline and at follow‐up visits.

**Conclusion:**

We report the existence of serum anti‐CRT Ab in IIM patients and demonstrate the possible association of anti‐CRT Ab with malignancy in IIM patients. Serum anti‐CRT Ab could serve as a novel candidate marker of cancer in IIM patients.

## Graphical abstract

To our knowledge, this is the first report to describe the prevalence and clinical significance of serum anti‐calreticulin autoantibodies (anti‐CRT Ab) in a large cohort of adult‐onset idiopathic inflammatory myopathy (IIM) patients. Serum anti‐CRT Ab could serve as a novel candidate marker of cancer in IIM patients, and detection of anti‐CRT Ab could be valuable in monitoring cancer recurrence in IIM patients with a history of solid tumors.

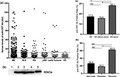

## Introduction

Idiopathic inflammatory myopathies (IIM), collectively known as myositis, are a heterogeneous group of systemic autoimmune disorders that are characterised by chronic skeletal muscle inflammation and related muscle weakness.^1^ Over the past years, a number of autoantibodies, traditionally defined as myositis‐specific or myositis‐associated autoantibodies (MSAs or MAAs) have been recognised in the sera of IIM patients. The close correlation between certain MSAs and distinct extramuscular manifestations contributes to the classification and management of IIM patients.^2^, ^3^ For instance, the risk of cancer is particularly high in recent‐onset adult dermatomyositis (DM) patients with autoantibodies against transcription intermediary factor 1γ (TIF‐1γ). In contrast, clinically amyopathic DM (ADM) patients with autoantibodies against melanoma differentiation‐associated protein 5 (MDA5) are more likely to develop rapidly progressive interstitial lung disease.^4^ However, the currently known MSAs and MAAs can only be detected in approximately 50% and 20% of IIM patients,^2^ respectively. This indicates that the remaining MSA/MAA‐negative patients may have potentially unrecognised autoantibodies. Therefore, discovering novel myositis autoantibodies and exploring their related clinical phenotypes are of great interest to both physicians and researchers.

Calreticulin (also known as CRT) is a multifunctional calcium‐binding chaperone protein, predominantly located in the endoplasmic reticulum. CRT has two major functions inside the endoplasmic reticulum: the modulation of Ca^2+^ homeostasis and the facilitation of protein folding, assembly and trafficking.^5^ The translocation of CRT to the cell membrane acts as an ‘eat‐me’ signal and contributes to the phagocytic uptake of dying, stressed and cancerous cells.^6^ Patients with a wide variety of cancers show abnormally upregulated expression levels of CRT in the sera and tumor tissues.^7^, ^8^ Further, CRT overexpression is associated with tumor invasion and poor prognosis in oesophageal cancers, gastric cancers and breast ductal carcinoma. Moreover, autoantibodies against CRT (anti‐CRT Ab) have been reported in the serum of patients with various solid tumors such as hepatic, colorectal, pancreatic, breast and bladder cancers.^9^, ^10^, ^11^, ^12^ Serum anti‐CRT Ab can also be detected in patients with systemic autoimmune diseases, including systemic lupus erythematosus (SLE), rheumatoid arthritis (RA), primary Sjogren’s syndrome (pSS) and inflammatory bowel disease.^13^, ^14^, ^15^, ^16^, ^17^, ^18^ The presence of anti‐CRT Ab in both the solid tumors and systemic autoimmune diseases suggests that they may play potential roles in anti‐tumor immune and autoimmune responses.

However, to the best of our knowledge, the association between anti‐CRT Ab and IIM has not yet been reported. Therefore, this study aimed to investigate the prevalence and clinical significance of anti‐CRT Ab in adult‐onset IIM patients and to evaluate its potential as a biomarker for cancer in IIM patients.

## Results

### Prevalence of serum anti‐CRT Ab in IIM patients

**Figure 1:**
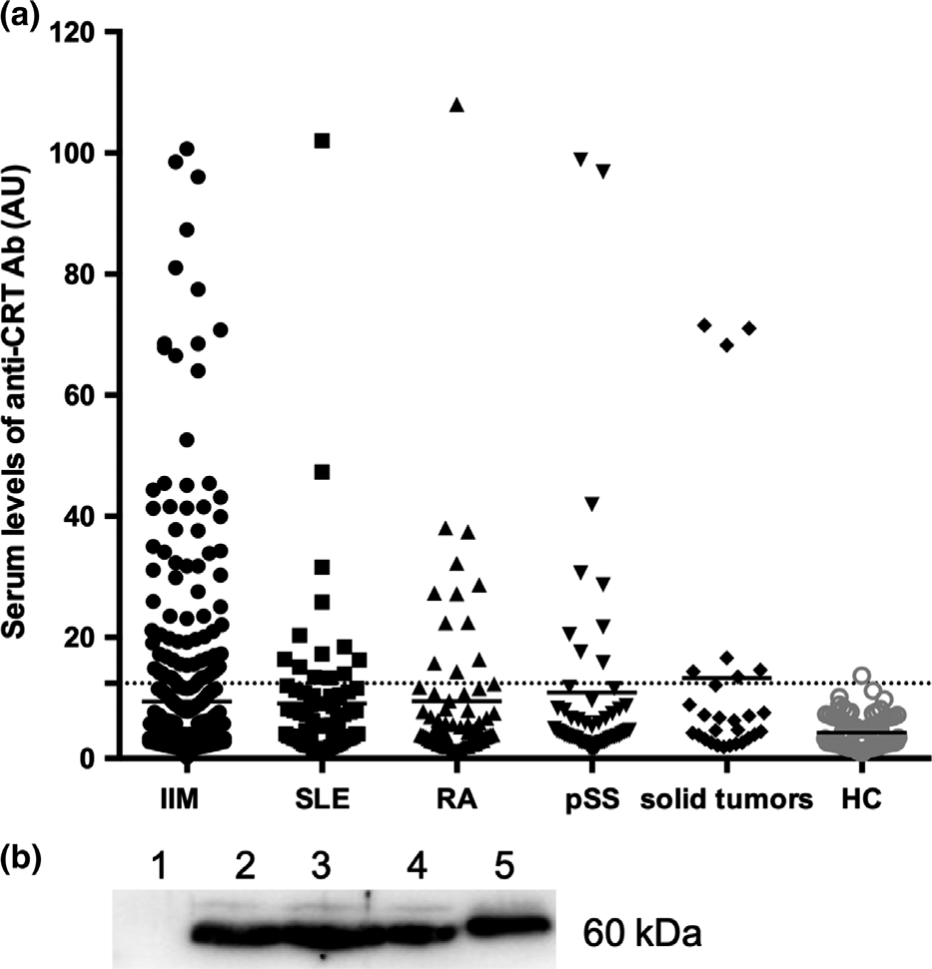
Detection of serum anti‐CRT Ab in IIM patients. **(a)** Serum anti‐CRT Ab was screened by ELISA in patients with various connective tissue diseases and solid tumors. The broken line indicates the cut‐off value calculated as the mean value of 81 HC samples plus a threefold standard deviation. The prevalence of anti‐CRT Ab in IIM (81/469, 17.3%), SLE (13/72, 18.1%), RA (12/70, 17.1%), pSS (9/54, 16.7%), solid tumors (7/28, 25%) and HC (1/81, 1.2%). **(b)** Patient sera were used to immunoprecipitate the CRT protein in K562 cell extracts. The immunoprecipitated bands represent the CRT protein bound by the antibody. Inputs used for immunoprecipitation include Lane 1, healthy control sera; Lanes 2‐4, anti‐CRT Ab‐positive sera from IIM patients, which immunoprecipated bands at a molecular weight of approximately 60 kDa; Lane 5, commercial rabbit polyclonal anti‐CRT antibody (Abcam, Cambridge, UK) as a positive control, which immunoprecipitated bands at a molecular weight of approximately 64 kDa. anti‐CRT Ab, anti‐calreticulin autoantibodies; ELISA, enzyme‐linked immunosorbent assay; IIM, idiopathic inflammatory myopathies; RA, rheumatoid arthritis; SLE, systemic lupus erythematosus; pSS, primary Sjogren’s syndrome; HC, healthy controls.

An enzyme‐linked immunosorbent assay (ELISA) using recombinant CRT protein was developed to screen for serum anti‐CRT Ab in patients with various connective tissue diseases (CTDs) and solid tumors. Based on the cut‐off level (mean value of healthy controls (HC) plus threefold standard deviation (SD)), 81 out of the 469 (17.3%) IIM patients were judged to be seropositive for anti‐CRT Ab (Figure 1a). Meanwhile, the SLE, RA, pSS, solid tumors patients and HC reached 18.1%, 17.1%, 16.7%, 25% and 1.2% positivity for anti‐CRT Ab, respectively. The prevalence of anti‐CRT Ab in IIM patients was significantly higher than that in HC (17.3% vs. 1.2%, *P* = 0.008), but was not significantly different to that in SLE, RA and pSS patients (all *P *> 0.05).

To confirm the presence of anti‐CRT Ab, the immunoprecipitation (IP) assay was performed in 35 of the 81 (43.2%) anti‐CRT Ab‐positive IIM patients. The 35 patients were selected because they had enough serum samples for the IP assay, while the remaining 46 patients lacked sera samples to carry out the IP assay. Immunoprecipitated bands at a molecular weight of approximately 60 kDa, corresponding to the CRT protein (lanes 2‐4, Figure 1b), were detected in ELISA‐positive sera. The immunoprecipitated bands were detected in 31 of 35 (88.5%) anti‐CRT Ab‐positive IIM patients. Interestingly, the commercial rabbit polyclonal anti‐CRT antibody (Abcam, Cambridge, UK) immunoprecipitated bands at a molecular weight of approximately 64 kDa (lane 5, Figure 1b).

### Characteristics of IIM patients with anti‐CRT Ab

The main demographic, clinical and laboratory features of the IIM patients with and without anti‐CRT Ab are summarised in Table 1. Of the 81 IIM patients with anti‐CRT Ab, 52 (64.2%), 10 (12.3%) and 19 (23.5%) were classified as DM, ADM and polymyositis (immune‐mediated necrotising myopathy) (PM (IMNM)), respectively. Seven IIM patients had isolated anti‐CRT Ab, and the remaining 74 patients had one or more concomitant MSA/MAAs. Malignancies more frequently occurred in IIM patients with anti‐CRT Ab than in patients without anti‐CRT Ab (22.2% vs. 11.9%, *P* = 0.013). There were no significant differences with respect to other clinical manifestations, such as muscle weakness, skin rashes or interstitial lung disease, between patients with and without anti‐CRT Ab. Furthermore, the prevalence of MSA/MAAs did not significantly differ between the two groups.

**Table 1:** Characteristics of IIM patients with and without anti‐CRT Ab

	Anti‐CRT Ab		
	Positive (*n* = 81)	Negative (*n* = 388)	*P*‐value
Female, *n* (%)	47 (58.0)	262 (67.5)	0.101
Age, years, mean ± SD	49.8 ± 16.3	48.6 ± 13.8	0.449
IIM subgroups, *n* (%)			
DM	52 (64.2)	246 (63.4)	0.892
ADM	10 (12.3)	43 (11.1)	0.744
PM (IMNM)	19 (23.5)	99 (25.5)	0.698
Disease duration, months, median (IQR)	6 (3‐22)	7 (3‐24)	0.484
Treatment‐naive, *n* (%)	29 (35.8)	116 (29.9)	0.302
Muscle weakness, *n* (%)	57 (70.4)	272 (70.1)	0.902
Interstitial lung disease, *n* (%)	40 (49.4)	208 (53.6)	0.488
Malignancy, *n* (%)	18 (22.2)	46 (11.9)	0.013
Heliotrope rash, *n* (%)	46 (56.8)	205 (52.8)	0.516
Gottron’s sign, *n* (%)	41 (50.6)	193 (49.7)	0.886
Mechanic hands, *n* (%)	22 (27.2)	137 (35.3)	0.159
Raynaud’s phenomenon, *n* (%)	2 (2.5)	17 (4.4)	0.628
Skin ulcer, *n* (%)	7 (8.6)	36 (9.3)	0.857
CK, median (IQR)	213 (48.5‐1575)	115 (45.2‐860)	0.183
LDH, median (IQR)	282 (208‐461)	271 (206.8‐407)	0.563
CRP, median (IQR)	0.497 (0.212‐1.210)	0.396 (0.184‐0.911)	0.189
ANA, *n* (%)	51 (62.9)	235 (60.6)	0.408
Occurrence of MSAs, *n* (%)			
MSA‐negative	15 (18.5)	99 (25.5)	0.182
Anti‐ARS	11 (13.6)	65 (16.8)	0.481
Anti‐TIF‐1γ	15 (18.5)	57 (14.7)	0.385
Anti‐MDA5	16 (19.8)	78 (20.1)	0.943
Anti‐NXP2	8 (9.9)	27 (6.9)	0.363
Anti‐Mi‐2	5 (6.2)	17 (4.4)	0.488
Anti‐SAE1	3 (3.7)	7 (1.8)	0.388
Anti‐SRP	5 (6.2)	25 (6.4)	0.928
Anti‐HMGCR	3 (3.7)	13 (3.3)	0.746
Occurrence of MAAs, *n* (%)			
Anti‐Ro‐52	23 (28.4)	90 (23.2)	0.308
Anti‐PM/Scl	4 (4.9)	16 (4.1)	0.762
Anti‐RNP	1 (1.2)	7 (1.8)	1.0
Anti‐Ku	2 (2.5)	8 (2.1)	0.686

ADM, amyopathic dermatomyositis; ANA, anti‐nuclear antibodies (titres ≥ 1:80 was defined as positive); anti‐ARS, anti‐aminoacyl tRNA synthetase antibodies including anti‐Jo‐1, anti‐PL‐7, anti‐PL‐12, anti‐OJ and anti‐EJ; anti‐CRT Ab, anti‐calreticulin autoantibodies; anti‐HMGCR, anti‐3‐hydroxy‐3‐methylglutaryl‐CoA reductase; anti‐MDA5, anti‐melanoma differentiation‐associated protein 5; anti‐Mi‐2, anti‐nucleosome remodelling deacetylase complex; anti‐NXP2, anti‐nuclear matrix protein 2; anti‐RNP, anti‐ribonucleoprotein; anti‐SAE1, anti‐small ubiquitin‐like modifier activating enzyme; anti‐SRP, anti‐signal recognition particle; anti‐TIF‐1γ, anti‐transcription intermediary factor 1γ; CK, creatine kinase; CRP, C‐reactive protein; DM, dermatomyositis; LDH, lactate dehydrogenase; MSA/MAA, myositis‐specific/myositis‐associated antibodies; PM (IMNM), polymyositis (immune‐mediated necrotising myopathy).

### Association between anti‐CRT Ab and malignancy in IIM patients

**Figure 2:**
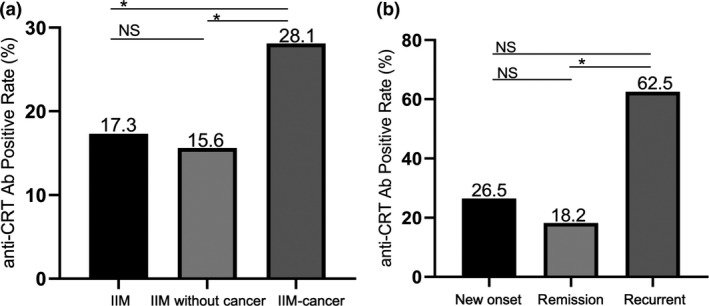
Association between serum anti‐CRT Ab with malignancy in IIM patients. **(a)** The prevalence of anti‐CRT Ab in IIM patients (81/469, 17.3%), IIM patients without cancer (63/405, 15.6%) and IIM–cancer patients (18/64, 28.1%). IIM patients with cancer had a higher prevalence of anti‐CRT Ab when compared to IIM patients (28.1% vs. 17.3%, *P* = 0.036) and IIM patients without cancer (28.1% vs. 15.6%, *P* = 0.013). **(b)** The prevalence of anti‐CRT Ab in IIM patients with new‐onset cancers (9/34, 26.5%), patients with cancers in remission (4/22, 18.2%) and patients with recurrent cancers (5/8, 62.5%). Patients with recurrent cancers presented a much higher prevalence of anti‐CRT Ab than patients with cancers in remission (62.5% vs. 18.2%, *P* = 0.032). anti‐CRT Ab, anti‐calreticulin autoantibodies; *P < 0.05; NS: not significant.

Since the IIM patients with anti‐CRT Ab had a higher prevalence of cancer, the association between anti‐CRT Ab and malignancy was further investigated. The prevalence of anti‐CRT Ab in the IIM–cancer patients was higher than that in the patients without cancer (28.1% vs. 15.6%, *P* = 0.013) (Figure 2a). Moreover, the prevalence of anti‐CRT Ab was distinct to each cancer status. The anti‐CRT Ab was detected positive in 9 (26.5%) patients with new‐onset cancers, 5 (62.5%) patients with recurrent cancers and 4 (18.2%) patients with cancers in remission. Patients with recurrent cancers had a much higher prevalence of anti‐CRT Ab than those with cancers in remission (62.5% vs. 18.2%, *P* = 0.032) (Figure 2b). However, the frequency of anti‐CRT Ab in the IIM patients with cancer in remission was similar to that in the patients without cancer (18.2% vs. 15.6%, *P* = 0.763). Although anti‐CRT Ab was also detected in the other CTD groups, we failed to observe a history of cancer or cancer occurrence in these anti‐CRT Ab‐positive SLE, RA and pSS patients during the observation period.

Subsequently, the characteristics of the IIM–cancer patients with anti‐CRT Ab were investigated. Of the 18 IIM–cancer patients with anti‐CRT Ab, 11(61.1%) were females, with a mean age of 58.9 years, and 14 (77.8%) were classified as DM patients (Table 2). All the patients had solid tumors, and the most common cancer types were lung, nasopharyngeal and breast (each *n* = 3), whereas the most common cancer types in the IIM–cancer patients without anti‐CRT Ab were ovarian (*n* = 9), followed by lung (*n* = 6) and thyroid (*n* = 6). Haematological malignancies were only observed in the anti‐CRT Ab‐negative patients. Furthermore, no significant difference was observed in the prevalence of MSA between the IIM–cancer patients with and without anti‐CRT Ab. In addition, the IIM–cancer patients with anti‐CRT Ab more frequently had recurrent cancers compared to those without anti‐CRT Ab (27.8% vs. 6.5%, *P* = 0.034) (Table 3). However, the association between the cancer status and autoantibody profile was not observed in the patients with anti‐TIF‐1γ Ab (all *P *> 0.05, Table 3). Thus, anti‐CRT Ab positivity may be linked with the occurrence of solid tumors and the recurrent status among IIM–cancer patients.

**Table 2:** General features of IIM–cancer patients with and without anti‐CRT Ab

	Anti‐CRT Ab		
	Positive (*n* = 18)	Negative (*n* = 46)	*P*‐value
Female, *n* (%)	11 (61.1)	31 (67.4)	0.634
Age, years, mean ± SD	58.9 ± 15.6	56.6 ± 11.4	0.516
Interval between cancer diagnosis and IIM diagnosis^a^, months, median (IQR)	7 (1‐15)	9 (3‐25)	0.459
IIM subgroups, *n* (%)			
DM	14 (77.8)	35 (76.1)	1.0
ADM	1 (5.5)	5 (10.9)	0.667
PM (IMNM)	3 (16.7)	6 (13.0)	0.703
Cancer types, *n* (%)			
Lung	3 (16.7)	6 (13.0)	0.703
Nasopharyngeal	3 (16.7)	3 (6.5)	0.338
Breast	3 (16.7)	4 (8.7)	0.391
Cervical	2 (11.1)	1 (2.2)	0.189
Oesophageal	2 (11.1)	1 (2.2)	0.189
Gastric	2 (11.1)	1 (2.2)	0.189
Ovarian	1 (5.6)	9 (19.6)	0.259
Thyroid	1 (5.6)	6 (13.0)	0.662
Bladder	1 (5.6)	0	‐
Lymphoma	0	4 (8.7)	‐
Colorectal	0	2 (4.3)	‐
Thymic	0	2 (4.3)	‐
Urothelial	0	1 (2.2)	‐
Bladder	0	1 (2.2)	‐
Gallbladder	0	1 (2.2)	‐
Laryngeal	0	1 (2.2)	‐
Choriocarcinoma	0	1 (2.2)	‐
Invasive mole	0	1 (2.2)	‐
Endometrial	0	1 (2.2)	‐
MSA occurrences, *n* (%)			
MSA‐negative	2 (11.1)	8 (17.4)	0.712
Anti‐ARS	1 (5.6)	5 (10.9)	0.667
Anti‐TIF‐1γ	11(61.1)	28 (60.9)	1.0
Anti‐NXP2	1 (5.6)	1 (2.2)	0.487
Anti‐MDA5	0	0	‐
Anti‐SAE1	1 (5.6)	1 (2.2)	0.487
Anti‐Mi‐2	0	1 (2.2)	‐
Anti‐SRP	1 (5.6)	2 (4.3)	1.0
Anti‐HMGCR	1 (5.6)	0	‐

ADM, amyopathic dermatomyositis; anti‐ARS, anti‐aminoacyl tRNA synthetase antibodies including anti‐Jo‐1, anti‐PL‐7, anti‐PL‐12, anti‐OJ and anti‐EJ; anti‐CRT Ab, anti‐calreticulin autoantibodies; anti‐HMGCR, anti‐3‐hydroxy‐3‐methylglutaryl‐CoA reductase; anti‐MDA5, anti‐melanoma differentiation‐associated protein 5; anti‐Mi‐2, anti‐nucleosome remodelling deacetylase complex; anti‐NXP2, anti‐nuclear matrix protein 2; anti‐SAE1, anti‐small ubiquitin‐like modifier activating enzyme; anti‐SRP, anti‐signal recognition particle; anti‐TIF‐1γ, anti‐transcription intermediary factor 1γ; DM, dermatomyositis; MSA, myositis‐specific antibodies; PM (IMNM), polymyositis (immune‐mediated necrotising myopathy).

^a^ Cancer was diagnosed either before or after an IIM diagnosis.

**Table 3:** Comparison of cancer status between patients with anti‐CRT Ab and patients with anti‐TIF‐1γ Ab

Cancer status, *n* (%)	Anti‐CRT Ab			Anti‐TIF‐1γ Ab		
	Positive (*n* = 18)	Negative (*n* = 46)	*P*‐value	Positive (*n* = 39)	Negative (*n* = 25)	*P*‐value
New‐onset	9 (50)	25 (54.4)	0.787	21 (53.8)	13 (52)	1.0
Recurrent	5 (27.8)	3 (6.5)	0.034	5 (12.8)	3 (12)	1.0
Remission	4 (22.2)	18 (39.1)	0.251	13 (33.4)	9 (36)	0.612

anti‐CRT Ab, anti‐calreticulin autoantibodies; anti‐TIF‐1γ Ab, anti‐transcription intermediary factor 1γ autoantibodies.

### Correlation between serum anti‐CRT Ab levels and disease activity in IIM patients

**Figure 3:**
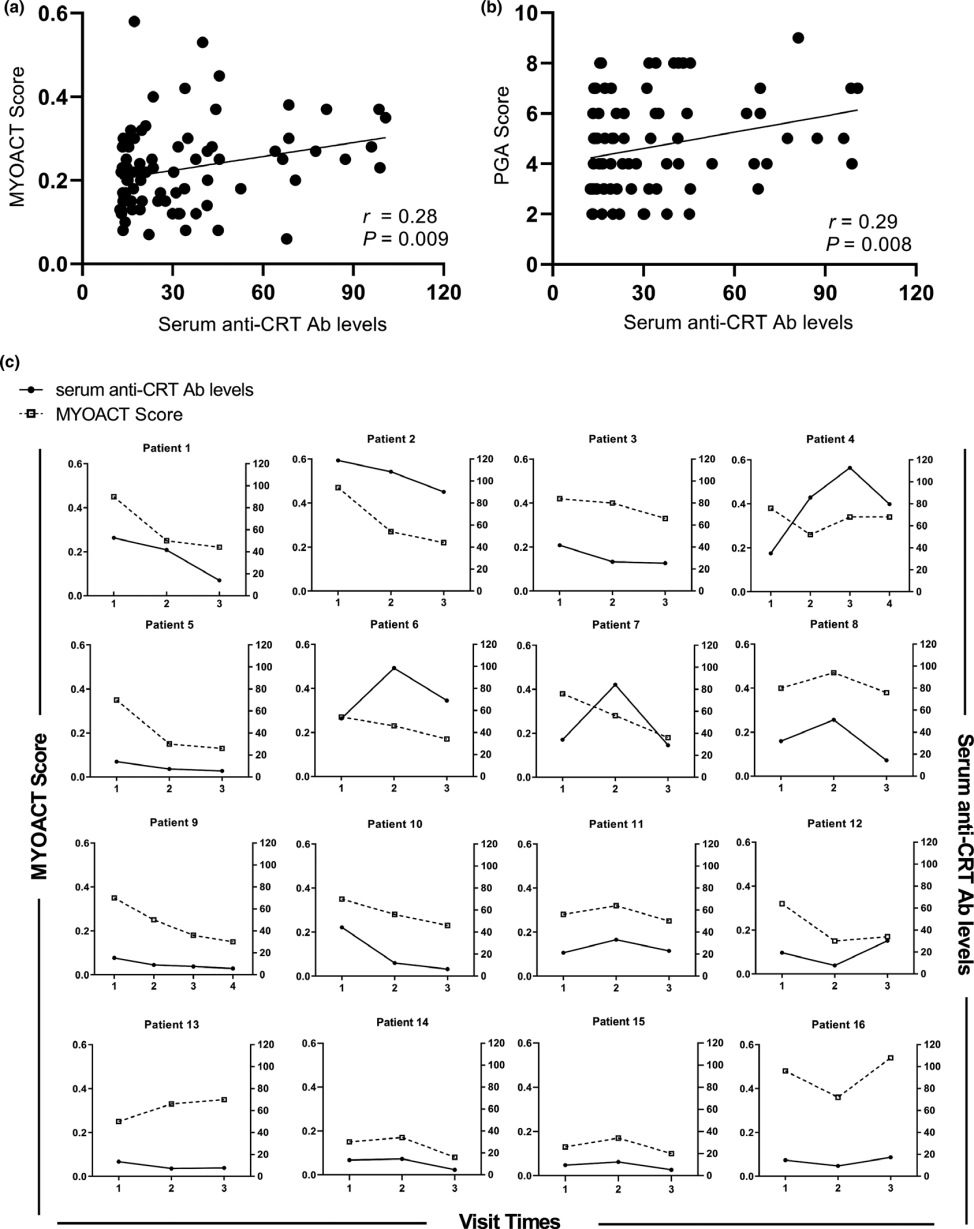
Positive correlations between serum anti‐CRT Ab levels and disease activity in IIM patients. **(a, b)** Cross‐sectional analyses of 81 anti‐CRT Ab‐positive IIM patients demonstrated a positive correlation between serum anti‐CRT Ab levels and MYOACT scores (Spearman *r* = 0.28, *P* = 0.009) and PGA scores (Spearman *r* = 0.29, *P* = 0.008). **(c)** Longitudinal analyses of 16 anti‐CRT Ab‐positive IIM patients demonstrated that the variations in anti‐CRT Ab titres positively correlated with the changes in MYOACT scores (*β* = 0.03, *P* < 0.001). Patient 14 to Patient 16 were IIM–cancer patients. anti‐CRT Ab, anti‐calreticulin autoantibodies; MYOACT, myositis disease activity assessment visual analogue scales; PGA, physician global assessment of disease activity.

Additionally, the relationship between serum anti‐CRT Ab levels and myositis disease activity was determined. The 81 IIM patients with anti‐CRT Ab were assessed for disease activity at baseline. Among them, 16 (including 3 IIM–cancer patients) were followed up, and their anti‐CRT Ab titres and disease activity were remeasured at subsequent visits. The cross‐sectional analyses of the 81 anti‐CRT Ab‐positive IIM patients revealed a weak positive correlation between the serum anti‐CRT Ab levels and the myositis disease activity assessment visual analogue scales (MYOACT) (Spearman’s *r* = 0.28, *P* = 0.009) and physician global assessment of disease activity (PGA) (Spearman’s *r* = 0.29, *P* = 0.008) (Figure 3a, b). The longitudinal analyses demonstrated that the variations in anti‐CRT Ab levels positively correlated with the changes in MYOACT scores during follow‐up visits (*β* = 0.03, *P* < 0.001) (Figure 3c).

## Discussion

Herein, we described the prevalence and related clinical phenotypes of serum anti‐CRT Ab‐positive patients in a large cohort of IIM patients. Approximately 17% of the IIM patients tested positive for anti‐CRT Ab, and malignancy more frequently occurred in the anti‐CRT Ab‐positive patients than in the anti‐CRT Ab‐negative patients (22.2% vs. 11.9%, *P* = 0.013). Moreover, the prevalence of anti‐CRT Ab differed in accordance with the cancer status. Patients with recurrent cancer had a much higher prevalence of anti‐CRT Ab than those with cancer in remission (62.5% vs. 18.2%, *P* = 0.032). Overall, we reported the presence of serum anti‐CRT Ab in IIM patients and revealed the possible association of anti‐CRT Ab with the occurrence of malignancy in IIM patients.

The molecular weight of human full‐length CRT is estimated to be 46‐kDa. However, CRT migrates on sodium dodecyl sulphate–polyacrylamide gel electrophoresis (SDS‐PAGE) at a molecular weight of about 60 to 65 kDa because of its acidic isoelectric point of 4.65 and high negative charge.^19^, ^20^ In this study, there was a difference in the immunoprecipitated band size between the IIM patients’ sera (60‐kDa) and the commercial rabbit polyclonal anti‐CRT antibody (64‐kDa). The manufacturers may use different fragments of peptides or full‐length proteins to immunise animals when they produce antibodies, and the antibodies produced by different manufacturers may recognise the same CRT protein with inconsistent molecular weight. Moreover, CRT post‐translational modifications may be different between the IIM patients and recombinant proteins. Therefore, these may be the reasons why the actual band size of the commercial anti‐CRT antibody differed from that of the IIM patient’s serum during electrophoresis.

Malignancies are one of the common and severe complications that can result in poor outcomes and high mortality in IIM patients.^21^, ^22^ The risk of developing cancer is increased in IIM patients carrying certain MSAs, such as the anti‐TIF‐1γ Ab, anti‐nuclear matrix protein 2 (NXP2) Ab or anti‐3‐hydroxy‐3‐methylglutaryl‐CoA reductase (HMGCR) Ab. However, malignancies can also occur in MSA‐negative patients.^23^, ^24^ Therefore, the identification of currently unrecognised autoantibodies for evaluating or predicting the occurrence of cancer in IIM patients remains an urgent and unmet clinical demand. The existence of anti‐CRT Ab was not specific to IIM patients as it was also detected in other CTD patients. However, we failed to observe the incidence of cancer in anti‐CRT Ab‐positive SLE, RA and pSS patients during the observation period; this finding was consistent with previous reports.^13^, ^14^, ^15^, ^16^, ^18^ In contrast, we demonstrated that anti‐CRT Ab was more prevalent in IIM patients with cancer than in IIM patients without cancer (28.1% vs. 15.6%, *P* = 0.013). Thus, anti‐CRT Ab could be a potential serological marker for cancer in IIM patients.

The strong association and close temporal relationship between anti‐TIF‐1γ Ab and the onset of cancer in adult DM patients have been well‐established.^25^, ^26^ A recent systematic review and meta‐analysis confirmed that anti‐TIF‐1γ Ab could be a valuable tool for diagnosing cancer‐associated DM, with a pooled sensitivity of 52%.^27^ In our study, the association between anti‐CRT Ab positivity and cancer occurrence was not as strong as that of anti‐TIF‐1γ Ab. However, we found that the patients with recurrent cancer had a much higher prevalence of anti‐CRT Ab than the patients with cancer in remission (62.5% vs. 18.2%, *P* = 0.032). Furthermore, we demonstrated that all the IIM–cancer patients with anti‐CRT Ab had solid tumors and were more likely to have recurrent cancers compared to the anti‐CRT Ab‐negative patients (27.8% vs. 6.5%, *P* = 0.034); whereas the correlation between the cancer status and autoantibody profile was not observed in the patients with anti‐TIF‐1γ Ab (all *P *> 0.05, Table 3). Our results indicated that, even though the sensitivity of anti‐CRT Ab for identifying cancer was not superior to that of anti‐TIF‐1γ Ab, the detection of anti‐CRT Ab in clinical practice may be valuable in monitoring cancer recurrence in IIM patients with a history of solid tumors.

CRT is a tumor‐associated antigen that plays a role in cancer development and invasion. Notably, the sera and tumor tissues of patients with various cancers have abnormally upregulated expression levels of CRT.^7^, ^8^ The CRT overexpression contributes to metastasis in oesophageal, gastric, pancreatic, prostate and breast cancers.^8^, ^28^ While the intracellular CRT overload can promote tumor progression, its translocation to the cell surface can induce an anti‐tumor immune response by priming immunogenic cell death. CRT expression on the cell surfaces is considered an ‘eat‐me’ signal that can be recognised by the CD91‐positive cells (mainly the dendritic cells and macrophages).^29^, ^30^ Collectively, these findings raise the possibility that the oncogenesis‐derived CRT may trigger the immune system to produce autoantibodies against CRT during the anti‐tumor response, which may contribute to the development of paraneoplastic myositis.^31^, ^32^

Little is known about the role of CRT in the pathogenesis of IIM. Two previous studies have demonstrated an increased expression of CRT in muscle biopsies of IIM patients. Vattemi *et al*. reported that the CRT expression was increased and it physically interacted with amyloid‐β precursor protein in the muscle fibres of inclusion body myositis (IBM) patients.^33^ Subsequently, Vitadello *et al*. reported that the CRT levels were higher in the muscle biopsies of myositis patients who tested positive for sarcolemmal MHC‐I staining than in healthy subjects and myositis patients who tested negative for both inflammation and MHC‐I staining.^34^ These findings suggested that CRT might exert its pathogenic effects on the development of myositis by enhancing the endoplasmic reticulum stress response and inducing the MHC‐I expression. In this study, we revealed a positive correlation between serum anti‐CRT Ab levels and myositis disease activity in cross‐sectional analyses. The longitudinal investigation on 16 anti‐CRT Ab‐positive IIM patients, including 3 IIM–cancer patients, demonstrated that the variations in anti‐CRT Ab levels positively correlated with the changes in MYOACT scores during the follow‐up visits. Our findings indicated that serum anti‐CRT Ab levels could serve as a marker of disease activity in IIM patients. As a consequence, longitudinally monitoring the serum anti‐CRT Ab levels may be useful for the assessment of disease course and treatment response in IIM patients.

There are several limitations to our study. Firstly, because of the limited number of cancer cases, further validation in larger cohorts is necessary to confirm the relationship between anti‐CRT Ab and malignancy in IIM. The utility of anti‐CRT Ab as a diagnostic marker for cancer in IIM needs additional studies before clinical application. Secondly, we could not exclude the possibility that some cancer cases may be lost to follow‐up and that the frequency of cancer may be underestimated in our IIM cohort since the median observation time was 22 months. Thirdly, the IP assays were not performed to validate the ELISA results of all enrolled IIM patients. The correlation for detection of anti‐CRT Ab between ELISA and IP should be determined in future studies. Last but not least, the prevalence and clinical significance of serum anti‐CRT Ab should be investigated in other subtypes of IIM, such as IBM, overlap myositis and juvenile myositis.

## Conclusions

In summary, we report the prevalence and clinical significance of anti‐CRT Ab in a large cohort of adult‐onset IIM patients and reveal the possible association of anti‐CRT Ab with the occurrence of malignancy in IIM patients. Serum anti‐CRT Ab could be a novel candidate marker of cancer in IIM. Furthermore, the detection of anti‐CRT Ab could be valuable in monitoring cancer recurrence in IIM patients with a history of solid tumors. Also, serum anti‐CRT Ab levels could serve as a potential marker of disease activity in IIM patients. Further studies are required to characterise the role of anti‐CRT Ab in the tumorigenesis and pathogenesis of IIM.

## Methods

### Patients

We enrolled 482 adult IIM patients (≥ 18 years of age at IIM diagnosis) who were admitted to the China‐Japan Friendship Hospital from 2012 to 2017. The diagnosis of IIM was based on the 1975 Bohan and Peter criteria.^35^, ^36^ Patients’ data, including demographics, clinical manifestations, laboratory results (e.g. levels of creatine kinase, lactate dehydrogenase, C‐reactive protein and anti‐nuclear antibodies) and muscle biopsy findings (if available), were retrospectively collected by reviewing the medical records. We screened the 482 patients following the 2017 European League Against Rheumatism/American College of Rheumatology (EULAR/ACR) classification criteria for IIM.^37^ Finally, the 469 IIM patients who fulfilled the 2017 EULAR/ACR classification criteria were included in this study. The 469 IIM patients were further subclassified into DM (*n* = 298), ADM (*n* = 53) and PM (IMNM) (*n* = 118) based on the classification tree.

IIM patients complicated with other CTDs (overlap myositis) were not classified in the 2017 EULAR/ACR classification criteria. Thus, we did not include overlap myositis as a subgroup of IIM patients in the current study. Because of the rarity of sporadic IBM cases in our cohort, it would not be possible to draw any valid conclusions from the findings. Therefore, we did not include IBM patients in this study.

The IIM patients routinely underwent cancer screening by physical examination, computed tomography, mammography, endoscopy and sometimes positron emission tomography/computed tomography at the time of initial hospitalisation. The occurrence of malignancy was monitored during follow‐up in our outpatient clinic, rehospitalisation and/or telephone interview. The observation period was defined as the interval between the myositis diagnosis and the last follow‐up. The median observation time was 22 months (interquartile range (IQR) 4‐37). By June 2019, we identified 64 (13.6%) IIM patients that also had a cancer diagnosis (IIM–cancer patients). The IIM–cancer patients were further divided into three groups based on their cancer status: new‐onset cancers (*n* = 34), cancers in remission (*n* = 22) and recurrent cancers (*n* = 8). ‘New‐onset cancers’ were defined as cancers that were initially diagnosed within a year of IIM diagnosis, but had not been treated at the time of blood sampling to detect anti‐CRT Ab. ‘Cancers in remission’ were defined as cancers that had been treated with surgical resection and/or standard chemotherapy, but showed no signs of recurrence within a year of enrolment. ‘Recurrent cancers’ were defined as cancers that had been treated with surgical resection and/or standard chemotherapy and showed specific signs of recurrence within a year of enrolment.

We included 196 patients with other CTDs (72 SLE, 70 RA and 54 pSS) as disease controls. These patients satisfied the 1997 ACR revised classification criteria for SLE,^38^ the 2010 EULAR/ACR classification criteria for RA^39^ and the 2002 American‐European Consensus Group classification criteria for pSS,^40^ respectively. The CTD patients who tested positive for anti‐CRT Ab were monitored for cancer occurrence, with a median observation time of 39 months (IQR 10‐74). Additionally, 28 patients with solid tumors (without CTDs) were included as disease controls. The sera of 28 patients with pretreated solid tumors, including lung, oesophageal and gastrointestinal cancers, were obtained from the Department of Thoracic Surgery and Department of Gastrointestinal Surgery. Eighty‐one HCs were enrolled and were age‐ and gender‐matched to the IIM cohort.

This study was approved by the Research Review Committee and the Ethical Review Committee of the China‐Japan Friendship Hospital under the registration number 2016‐117. Informed consent was obtained from all the patients in accordance with the Declaration of Helsinki.

### Serum collection and MSA/MAA detection

Serum samples were collected from IIM patients at initial visit and at subsequent visits and then stored at −80°C until the analyses. The sera of 81 HC were obtained from the Department of Health Examination. Serum MSA/MAAs against TIF‐1γ, MDA5, NXP2, Mi‐2 (α and β), SAE1, SRP, Jo‐1, PL‐7, PL‐12, EJ, OJ, Ro‐52, PM/Scl (75 and 100), RNP and Ku were detected using a commercial immunoblot assay (EUROIMMUN, Luebeck, Germany). The assay was performed and reported manually following the manufacturer’s instructions. The anti‐HMGCR Ab was detected using a commercial ELISA kit (Inova Diagnostics, San Diego, CA, USA) performed according to the manufacturer’s instructions.

### Development of ELISA system for detecting anti‐CRT Ab

An ELISA system was developed to detect anti‐CRT Ab as previously described,^10^ with some modifications. Briefly, 96‐well flat microtitre plates (Thermo Fisher Scientific, Waltham, MA, USA) were coated with recombinant human CRT protein (OriGene Technologies, Rockville, MD, USA) at 50 ng per well in pH 9.6 carbonate buffer and the plates were then incubated overnight at 4°C. After 3 washes with phosphate‐buffered saline containing 0.05% Tween‐20 (PBST), the plates were coated with the blocking buffer (5% skim milk in PBS) for 2 h at room temperature (RT). After 3 washes with PBST, the serum samples (diluted at 1:100 with 1% bovine serum albumin in PBST) were added to the wells in duplicates and followed by incubation for 2 h at RT. After 4 washes with PBST, horseradish peroxidase‐conjugated anti‐human immunoglobulin G (Abcam, Cambridge, UK) was added to the wells and incubated for 1 h at RT. After 4 washes with PBST, the immunoreactivity was visualised by adding tetramethylbenzidine (Solarbio, Beijing, China). After incubation for 15 min at RT, the reaction was stopped by adding 2 m sulphuric acid (Solarbio, Beijing, China), and the optical density at 450 nm was measured using a microplate reader (Bio‐Rad Laboratories, Richmond, CA, USA). A serum sample with immunoprecipitation‐validated anti‐CRT Ab was used to prepare a standard curve consisting of 0, 2, 4, 8, 16, 32, 64 and 128 Units (U).^41^

### Immunoprecipitation assay

The IP assay was performed as previously described,^31^ with some modifications. Briefly, 10 μL of a serum sample, extracts from 10^6^ K562 cells and Nonidet P40 (NP‐40) lysis buffer (Beyotime, Shanghai, China) were homogenised in a tube (500 μL of total volume), and incubated overnight at 4°C. Then, 30 μL of protein A agarose (Roche Diagnostics, Mannheim, Germany) prewashed with PBS was added into the mix, followed by incubation for 3 h at 4°C. After 5 washes with the IP buffer (0.5% NP‐40 in PBS), the agarose bead‐bound proteins were mixed with loading buffer and denatured at 100°C for 5 min. After centrifugation (12 000 *g* at 4°C, 10 min), the supernatant was harvested and electrophoresed by SDS‐PAGE, and then transferred to the Western blotting membranes. After 2 washes with Tris‐buffered saline containing 0.05% Tween‐20 (TBST), the membranes were incubated in the blocking buffer (5% skim milk in TBST) for 2 h at RT. Next, these membranes were reacted with rabbit polyclonal anti‐CRT antibodies (dilution 1:1000; Abcam, Cambridge, UK) overnight at 4°C. After 3 washes, the membranes were incubated with horseradish peroxidase‐conjugated goat anti‐rabbit immunoglobulin antibodies (dilution 1:5000; Abcam, Cambridge, UK) for 1 h at RT. The signals were developed using the Super Signal Maximum Sensitivity Substrate (Thermo Fisher Scientific, Waltham, MA, USA).

### Assessment of disease activity

Eighty‐one IIM patients with anti‐CRT Ab were assessed for disease activity at baseline. Of the 81 IIM patients, 16 were randomly chosen and were followed up for 2‐3 times with serum samples collected at the time of follow‐up. Disease activity was assessed at follow‐up visits. Disease activity including the PGA and MYOACT was evaluated according to the International Myositis Assessment and Clinical Studies Group core set measures.^42^, ^43^ Disease activity was evaluated by two physicians (GYP, LX) independently, who were blinded to the serum anti‐CRT Ab levels.

### Statistical analyses

The categorical variables are presented as count and percentages, while the continuous variables are presented as mean with SD or medians with IQR. For continuous variables, data were compared using the Mann–Whitney *U*‐test for non‐normal distributed samples. For categorical variables, data were compared using the chi‐squared or Fisher’s exact tests. The cross‐sectional correlation analyses between anti‐CRT Ab levels and disease activity were conducted using the Spearman’s rank correlation coefficients, while the longitudinal data were analysed using the generalised estimating equations. The statistical analyses were carried out using SPSS (version 23.0, IBM Corp, Armonk, N.Y., USA), and the figures were plotted using Prism (version 6.0, GraphPad, San Diego, CA, USA). A *P*‐value (two‐sided) < 0.05 was considered statistically significant.

## Conflict of interest

The authors declare no conflict of interest.

## Author contribution

**He Chen:** Conceptualization; Data curation; Formal analysis; Investigation; Methodology; Project administration; Writing‐original draft; Writing‐review & editing. **Heng Yang:** Conceptualization; Investigation; Methodology; Resources. **Qiu‐Xiang Cheng:** Investigation; Methodology; Resources. **Yong‐Peng Ge:** Data curation; Investigation. **Qing‐Lin Peng:** Data curation; Formal analysis; Methodology. **Ya‐Mei Zhang:** Methodology. **Gen‐Hong Cheng:** Funding acquisition; Resources. **Guo‐Chun Wang:** Data curation; Funding acquisition; Writing‐review & editing. **Xin Lu:** Conceptualization; Data curation; Funding acquisition; Project administration; Supervision; Writing‐review & editing.
